# Supplementary material for: The Efficacy of Transcranial Direct Current Stimulation in Enhancing Surgical Skill Acquisition: A Preliminary Meta-Analysis of Randomized Controlled Trials
Source: Brain Sci. 2021 May 27;11(6):707. doi: 10.3390/brainsci11060707 (PMC8229080; doi:10.3390/brainsci11060707)
Supplement: Supplementary file 1 [file brainsci-11-00707-s001.zip › All supplement figures 20210525.pptx]

## Slide 1
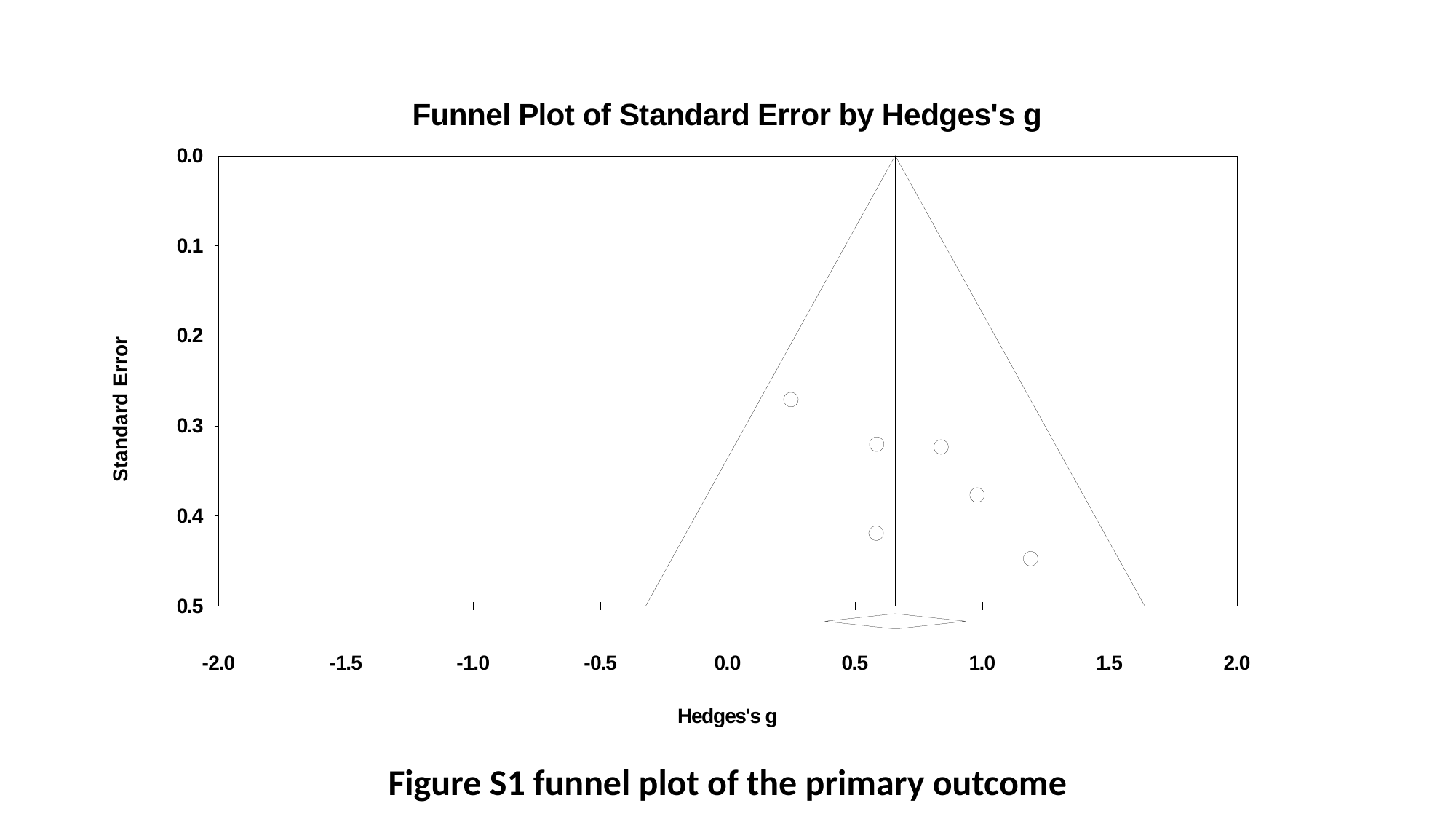

Figure S1 funnel plot of the primary outcome

## Slide 2
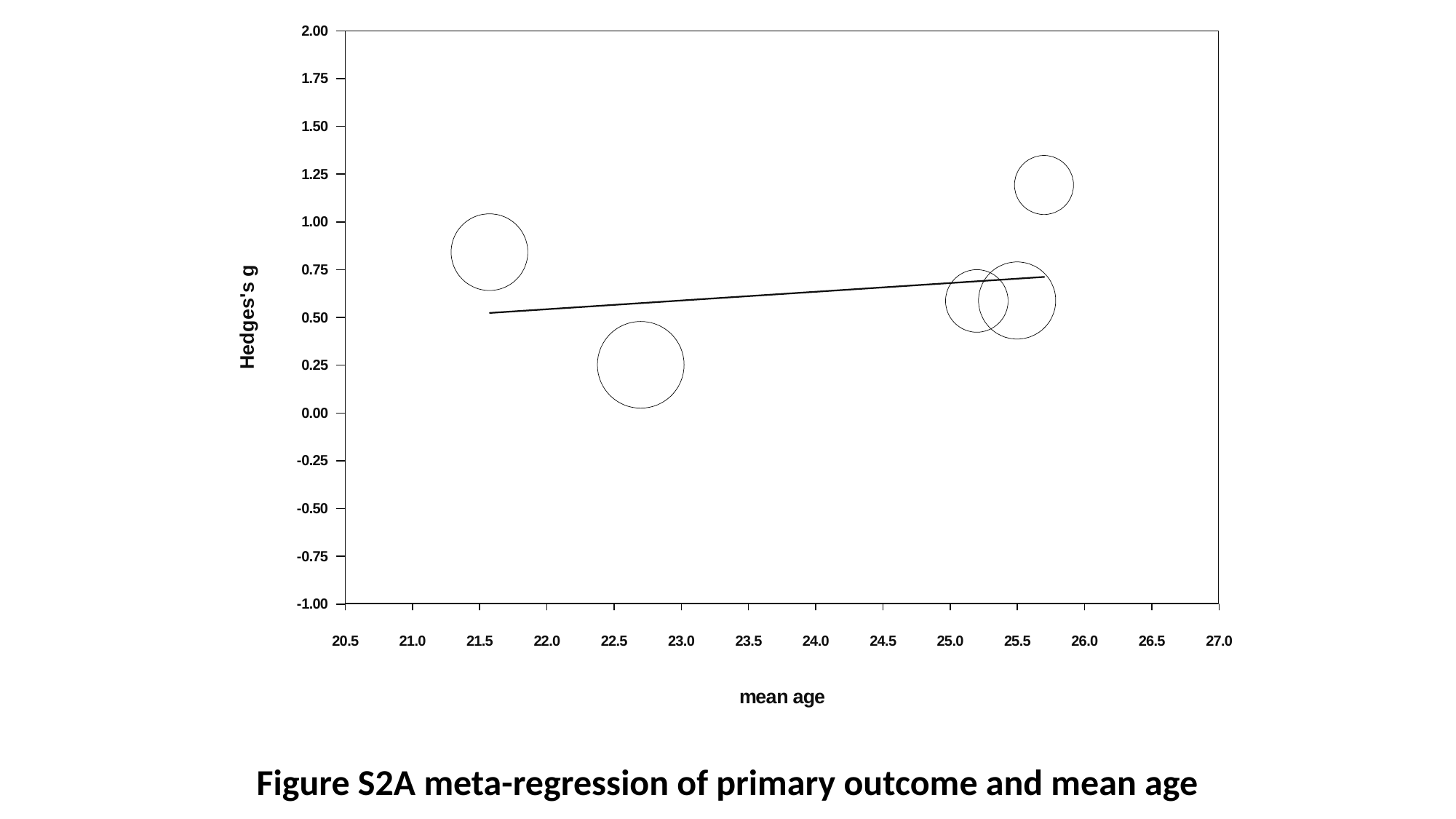

Figure S2A meta-regression of primary outcome and mean age

## Slide 3
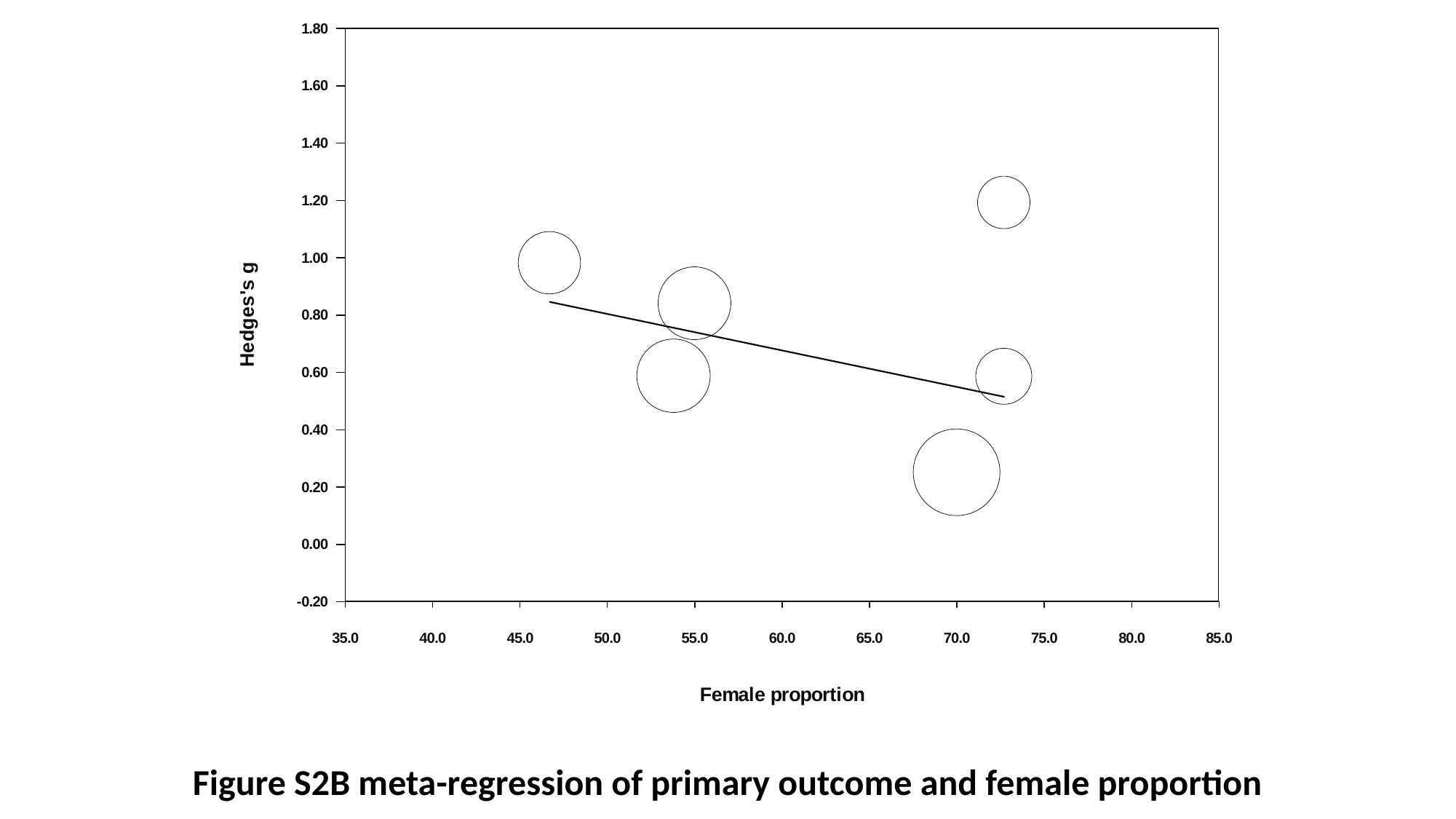

Figure S2B meta-regression of primary outcome and female proportion

## Slide 4
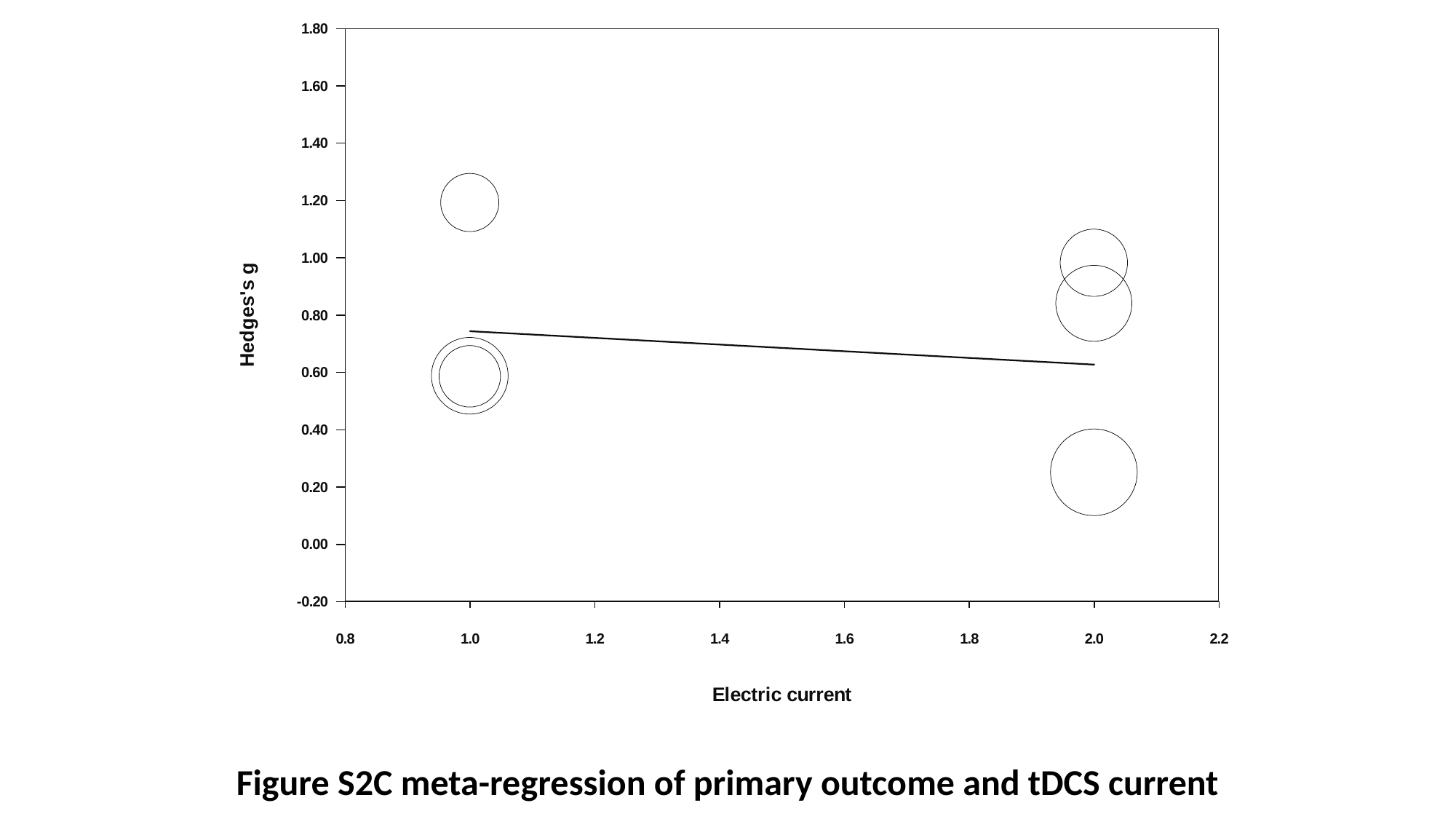

Figure S2C meta-regression of primary outcome and tDCS current

## Slide 5
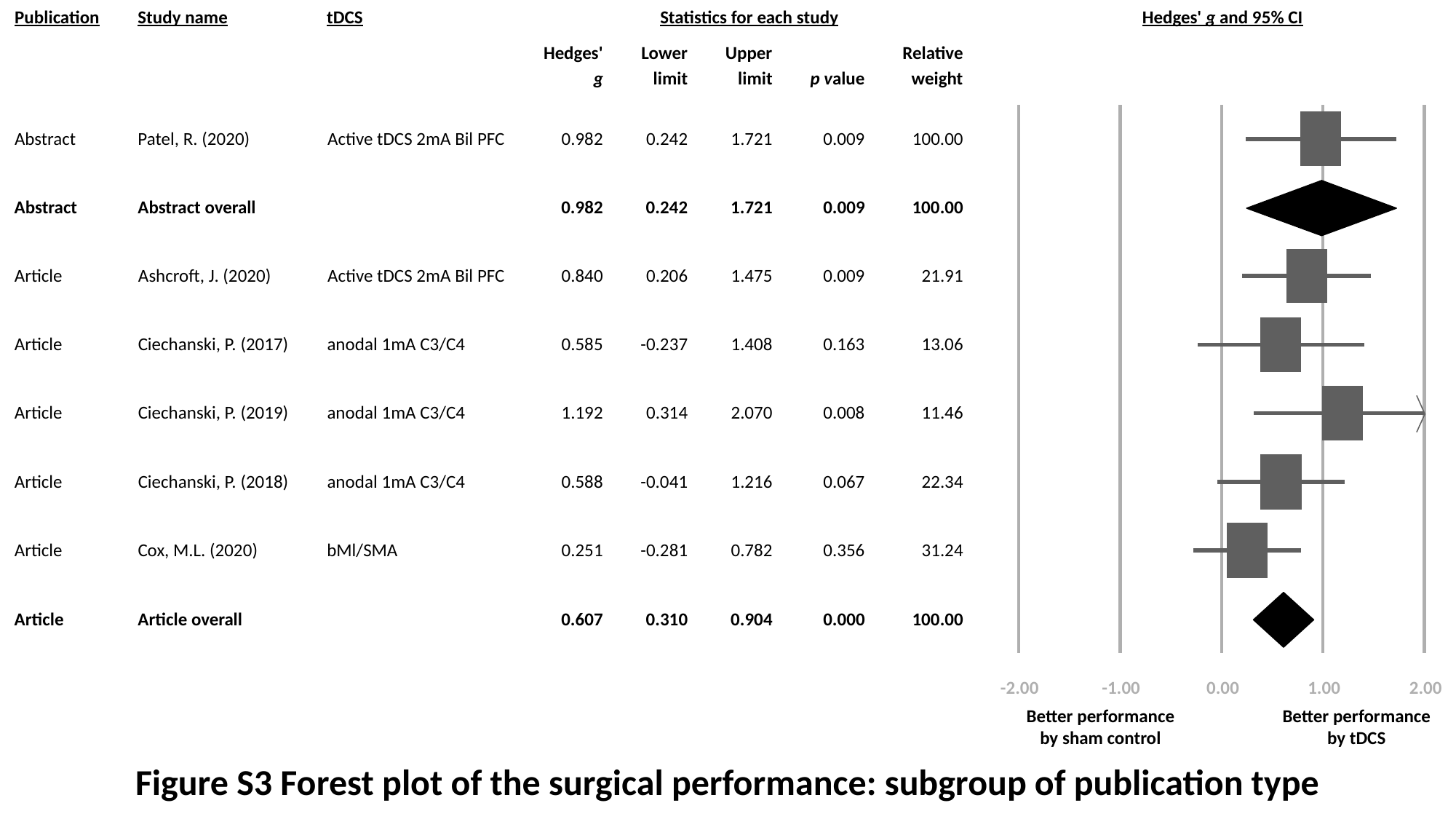

Publication
Study name
tDCS
Statistics for each study
Hedges' g and 95% CI
Hedges'
Lower
Upper
Relative
g
limit
limit
p value
weight
Abstract
Patel, R. (2020)
Active tDCS 2mA Bil PFC
0.982
0.242
1.721
0.009
100.00
Abstract
Abstract overall
0.982
0.242
1.721
0.009
100.00
Article
Ashcroft, J. (2020)
Active tDCS 2mA Bil PFC
0.840
0.206
1.475
0.009
21.91
Article
Ciechanski, P. (2017)
anodal 1mA C3/C4
0.585
-0.237
1.408
0.163
13.06
Article
Ciechanski, P. (2019)
anodal 1mA C3/C4
1.192
0.314
2.070
0.008
11.46
Article
Ciechanski, P. (2018)
anodal 1mA C3/C4
0.588
-0.041
1.216
0.067
22.34
Article
Cox, M.L. (2020)
bMl/SMA
0.251
-0.281
0.782
0.356
31.24
Article
Article overall
0.607
0.310
0.904
0.000
100.00
-2.00
-1.00
0.00
1.00
2.00
Better performance by tDCS
Better performance by sham control
Figure S3 Forest plot of the surgical performance: subgroup of publication type

## Slide 6
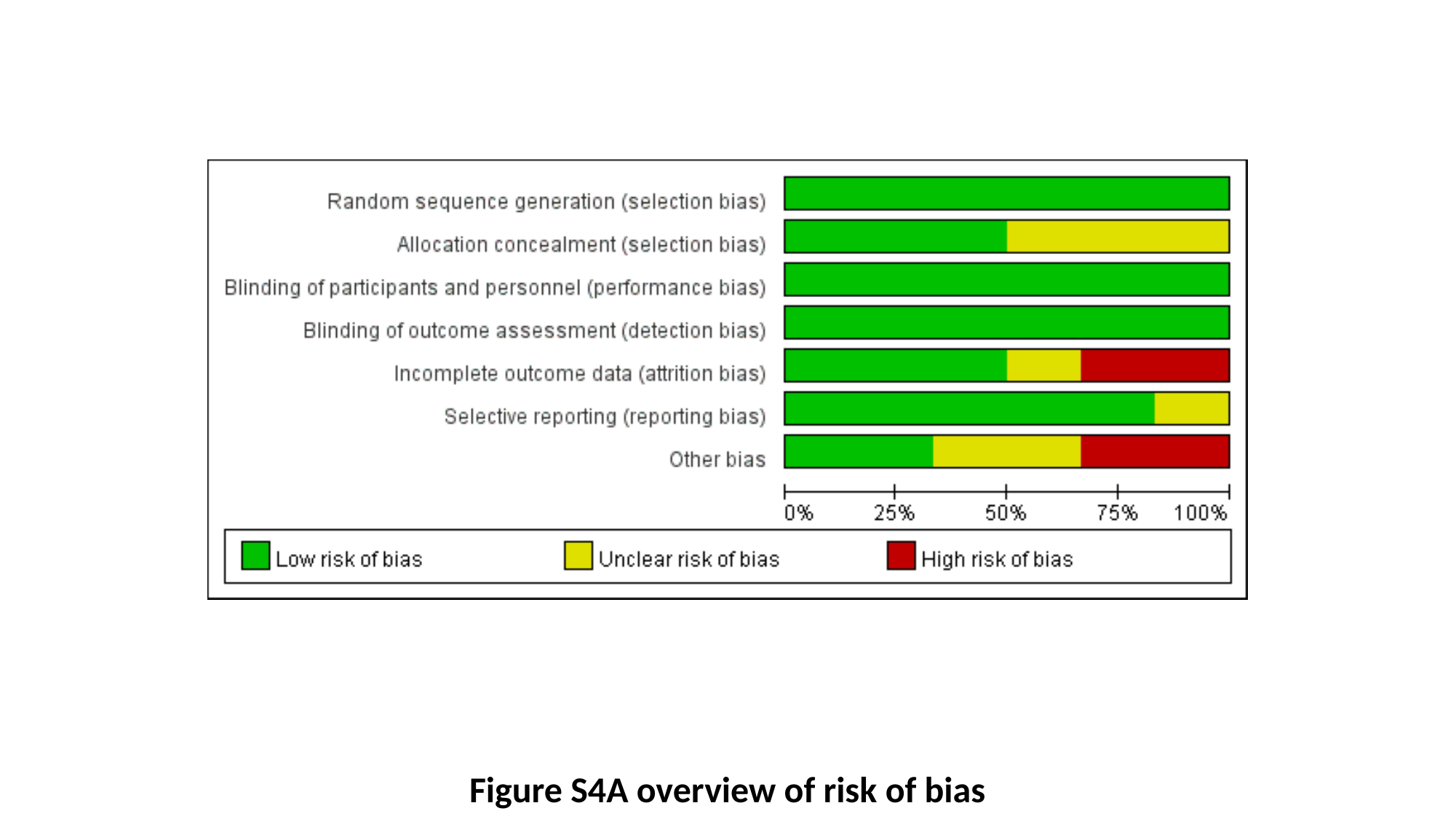

Figure S4A overview of risk of bias

## Slide 7
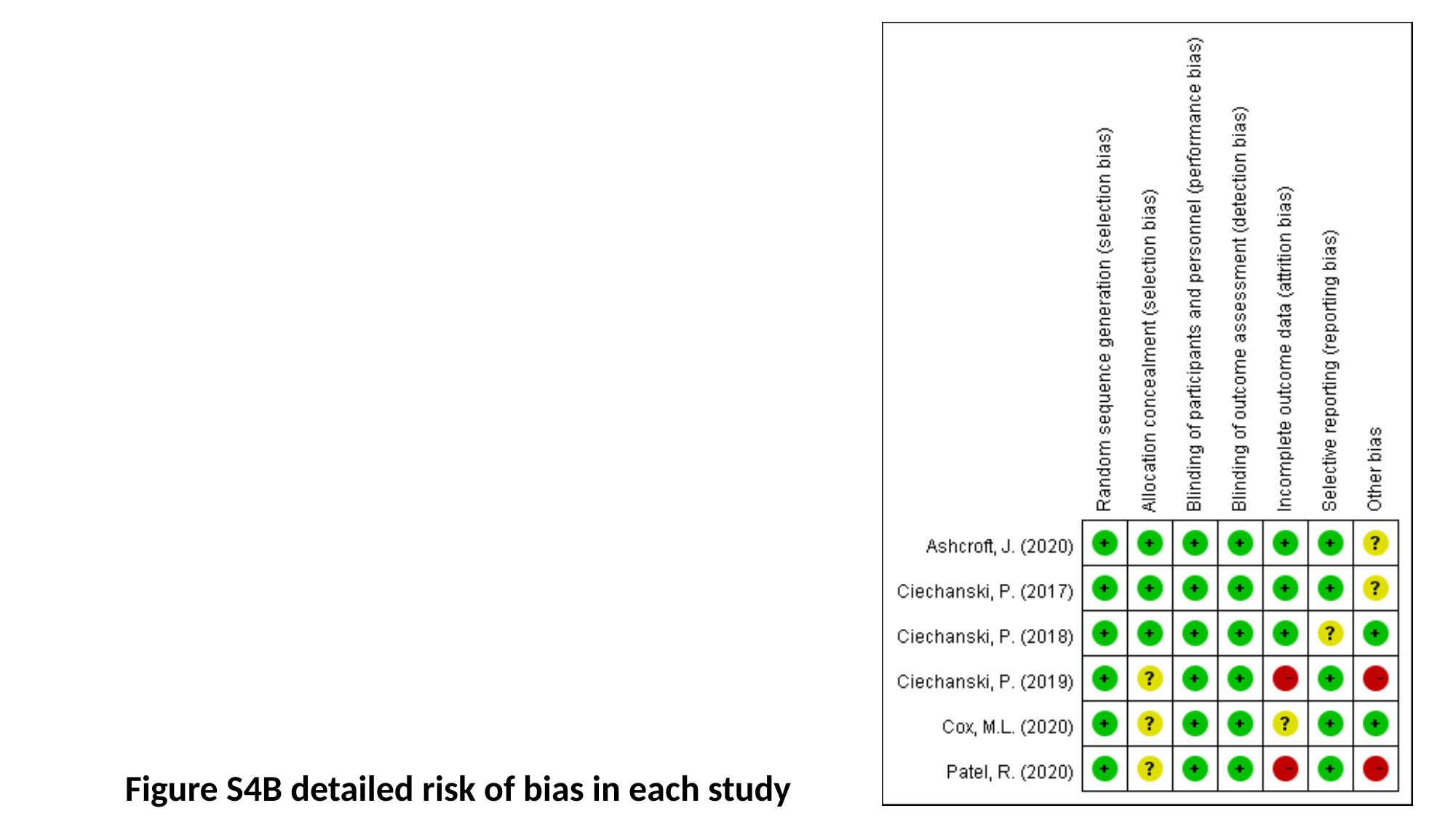

Figure S4B detailed risk of bias in each study
