# Supplementary material for: The Efficacy of Transcranial Direct Current Stimulation in Enhancing Surgical Skill Acquisition: A Preliminary Meta-Analysis of Randomized Controlled Trials
Source: Brain Sci. 2021 May 27;11(6):707. doi: 10.3390/brainsci11060707 (PMC8229080; doi:10.3390/brainsci11060707)
Supplement: Supplementary file 1 [file brainsci-11-00707-s001.zip › All Supplement table 20210524.docx]

**Supplementary Methods and Materials**

**General guideline**

We followed the instructions of the latest version of PRISMA 2020 guidelines (eTable 1) [1] and AMSTAR 2 (Assessing the methodological quality of systematic reviews) Guidelines [2]. The current study received approval from the Institutional Review Board of the Tri-Service General Hospital (TSGHIRB: B-109-29).

**Database searches and identification of eligible papers**

Two authors made independent electronic searches in the PubMed, Embase, ClinicalKey, Cochrane CENTRAL, ProQuest, ScienceDirect, Web of Science, and ClinicalTrials.gov platforms with keyword of (deep transcranial magnetic stimulation OR dTMS OR repetitive transcranial magnetic stimulation OR rTMS OR TMS OR non-invasive brain stimulation OR theta burst stimulation OR transcranial direct current stimulation OR TBS OR tDCS OR vagus nerve stimulation OR vagal nerve stimulation OR tVNS OR nVNS OR VNS OR static magnetic field stimulation) AND (skill OR professionalism OR skill acquisition) AND (surgery OR surgical OR surgeon) AND (random OR randomized OR randomised) through April 19^th^, 2021 (the detailed search strategy is provided in eTable 2). In the initial stage, these two authors screened the titles and abstracts for eligibility through consensus. Later, a third reviewer was consulted in situations in which the two authors could not achieve consensus. Furthermore, to expand the pool of potential studies, we consulted the reference lists of review articles and performed further manual searches [3-5]. No language restriction had been applied.

**Inclusion and exclusion criteria**

The PICO (population, intervention, comparison, outcome) setting of the current meta-analysis included: (1) P: participants receiving surgical skill training; (2) I: transcranial direct-current stimulation (tDCS); (3) C: sham stimulation; and (4) O: the change in surgical performance. To generate a recruited study list, the following inclusion criteria were used: (1) randomized controlled trials (RCTs) investigating the difference in changes of surgical performance after tDCS or sham stimulation, (2) RCTs of either sham-control or active-control, and (3) RCTs investigating different efficacies in surgical skill training with the tDCS intervention.

The exclusion criteria were: (1) studies that were not RCTs, and (2) studies that did not investigate the difference in changes of surgical performance after tDCS and sham stimulation.

**Methodological quality appraisal**

To investigate the methodological quality of recruited studies, we used the Cochrane risk-of-bias tool, which consisted of seven main items (randomization, concealment, blindness of participants, blindness of investigator, attrition bias, reporting bias, and other bias) [6].

**Primary outcomes**

The primary outcome was the change in surgical performance associated with NIBS or sham stimulation. Because the definition of surgical performance varied widely among the individual RCTs, we did not specifically set a limitation to the specific definition of surgical performance. Rather, we chose the primary outcome of surgical performance applied in each RCT to represent the primary findings of each RCT.

**Secondary outcomes and safety profile**

Secondary outcomes were the changes in error scores. The error scores were defined as improper transfers in laparoscopic training or resection of healthy brain in neurosurgical training. Safety profile was defined as the rate of local discomfort (i.e., itching, tingling pain, or erythematous).

**Data extraction and management**

Two independent authors extracted data from the recruited studies, including primary and secondary outcomes and safety profiles. In situations where data were unavailable in the literature, we tried to electronically contact the corresponding authors to request original data; this was done on at least two different occasions. In situations of duplicated data, we choose the largest dataset to include in the current meta-analysis.

**Meta-analysis**

Based on the presumed heterogeneous selection population among all the recruited studies, the current meta-analysis was conducted with random-effects meta-analysis models. The meta-analysis procedure was performed with Comprehensive Meta-Analysis software, version 3 (Biostat, Englewood, NJ). We chose Hedges’ *g* and 95% confidence intervals (95%CIs) as the main effect sizes (ESs) of the primary and secondary outcomes. We chose odds ratios and 95% CIs as the ESs of the safety profiles. If the two-tailed *p* values were less than 0.05, we considered the differences to be “statistically significant”.

**Heterogeneity, publication bias, and sensitivity test**

To detect potential heterogeneity, we used the Q statistic and corresponding *p* values [7]. At the same time, to evaluate the potential publication bias, we visually inspected funnel plots when there were fewer than 10 datasets [8] or performed Egger’s regression tests when there were 10 or more datasets [9]. We used the Duval and Tweedie trim-and-fill test when there was evidence of publication bias [10]. A sensitivity test was performed using the one study removal method, in which one study was excluded from the analyses at a time to observe whether significant or insignificant results of the meta-analyses were biased by outliers [11].

**Meta-regression and subgroup meta-analysis**

We used the meta-regression procedure and subgroup meta-analyses to discover the potential sources of heterogeneity and confounding factors. Specifically, in situations of at least three sets of datasets, we performed the meta-regression procedure using the unrestricted maximum likelihood method. In addition, we performed a subgroup meta-analysis according to the cortex at which the NIBS was targeted. In addition, we examined the differences in the ESs of individual subgroups with interaction tests [12].

**Reference:**

1. Page, M.J.; McKenzie, J.E.; Bossuyt, P.M.; Boutron, I.; Hoffmann, T.C.; Mulrow, C.D.; Shamseer, L.; Tetzlaff, J.M.; Akl, E.A.; Brennan, S.E.; et al. The PRISMA 2020 statement: an updated guideline for reporting systematic reviews. *Bmj* **2021**, *372*, n71, doi:10.1136/bmj.n71.

2. Shea, B.J.; Reeves, B.C.; Wells, G.; Thuku, M.; Hamel, C.; Moran, J.; Moher, D.; Tugwell, P.; Welch, V.; Kristjansson, E.; et al. AMSTAR 2: a critical appraisal tool for systematic reviews that include randomised or non-randomised studies of healthcare interventions, or both. *Bmj* **2017**, *358*, j4008, doi:10.1136/bmj.j4008.

3. Gao, Y.; Cavuoto, L.; Schwaitzberg, S.; Norfleet, J.E.; Intes, X.; De, S. The Effects of Transcranial Electrical Stimulation on Human Motor Functions: A Comprehensive Review of Functional Neuroimaging Studies. *Front Neurosci* **2020**, *14*, 744, doi:10.3389/fnins.2020.00744.

4. Lefaucheur, J.P.; Antal, A.; Ayache, S.S.; Benninger, D.H.; Brunelin, J.; Cogiamanian, F.; Cotelli, M.; De Ridder, D.; Ferrucci, R.; Langguth, B.; et al. Evidence-based guidelines on the therapeutic use of transcranial direct current stimulation (tDCS). *Clin Neurophysiol* **2017**, *128*, 56-92, doi:10.1016/j.clinph.2016.10.087.

5. Patel, R.; Ashcroft, J.; Darzi, A.; Singh, H.; Leff, D.R. Neuroenhancement in surgeons: benefits, risks and ethical dilemmas. *Br J Surg* **2020**, *107*, 946-950, doi:10.1002/bjs.11601.

6. Higgins, J.; Green, S. *Cochrane Handbook for Systematic Reviews of Interventions Version 5.0.2*; The Cochrane Collaboration: 2009.

7. Higgins, J.P.; Thompson, S.G.; Deeks, J.J.; Altman, D.G. Measuring inconsistency in meta-analyses. *Bmj* **2003**, *327*, 557-560, doi:10.1136/bmj.327.7414.557.

8. Higgins, J.P.; Green, S. 10.4.3.1 Recommendations on testing for funnel plot asymmetry. *Cochrane Handbook for Systematic Reviews of Interventions* **2011**.

9. Egger, M.; Davey Smith, G.; Schneider, M.; Minder, C. Bias in meta-analysis detected by a simple, graphical test. *Bmj* **1997**, *315*, 629-634.

10. Duval, S.; Tweedie, R. Trim and fill: A simple funnel-plot-based method of testing and adjusting for publication bias in meta-analysis. *Biometrics* **2000**, *56*, 455-463.

11. Tobias, A. Assessing the influence of a single study in meta-analysis. *Stata Tech Bull* **1999**, *47*, 15–17.

12. Altman, D.G.; Bland, J.M. Interaction revisited: the difference between two estimates. *Bmj* **2003**, *326*, 219.

**Table S1:** Checklist of PRISMA guideline

| **Section and Topic** | **Item #** | **Checklist item** | **Page where item is reported** |
| --- | --- | --- | --- |
| **TITLE** | | |  |
| Title | 1 | Identify the report as a systematic review. | 1 |
| **ABSTRACT** | | |  |
| Abstract | 2 | See the PRISMA 2020 for Abstracts checklist. | 4 |
| **INTRODUCTION** | | |  |
| Rationale | 3 | Describe the rationale for the review in the context of existing knowledge. | 5-6 |
| Objectives | 4 | Provide an explicit statement of the objective(s) or question(s) the review addresses. | 5-6 |
| **METHODS** | | |  |
| Eligibility criteria | 5 | Specify the inclusion and exclusion criteria for the review and how studies were grouped for the syntheses. | 7-9, Appendix |
| Information sources | 6 | Specify all databases, registers, websites, organisations, reference lists and other sources searched or consulted to identify studies. Specify the date when each source was last searched or consulted. | 7-9, Appendix |
| Search strategy | 7 | Present the full search strategies for all databases, registers and websites, including any filters and limits used. | 7-9, Appendix |
| Selection process | 8 | Specify the methods used to decide whether a study met the inclusion criteria of the review, including how many reviewers screened each record and each report retrieved, whether they worked independently, and if applicable, details of automation tools used in the process. | 7-9, Appendix |
| Data collection process | 9 | Specify the methods used to collect data from reports, including how many reviewers collected data from each report, whether they worked independently, any processes for obtaining or confirming data from study investigators, and if applicable, details of automation tools used in the process. | 7-9, Appendix |
| Data items | 10a | List and define all outcomes for which data were sought. Specify whether all results that were compatible with each outcome domain in each study were sought (e.g. for all measures, time points, analyses), and if not, the methods used to decide which results to collect. | 7-9, Appendix |
|  | 10b | List and define all other variables for which data were sought (e.g. participant and intervention characteristics, funding sources). Describe any assumptions made about any missing or unclear information. | 7-9, Appendix |
| Study risk of bias assessment | 11 | Specify the methods used to assess risk of bias in the included studies, including details of the tool(s) used, how many reviewers assessed each study and whether they worked independently, and if applicable, details of automation tools used in the process. | 7-9, Appendix |
| Effect measures | 12 | Specify for each outcome the effect measure(s) (e.g. risk ratio, mean difference) used in the synthesis or presentation of results. | 7-9, Appendix |
| Synthesis methods | 13a | Describe the processes used to decide which studies were eligible for each synthesis (e.g. tabulating the study intervention characteristics and comparing against the planned groups for each synthesis (item #5)). | 7-9, Appendix |
|  | 13b | Describe any methods required to prepare the data for presentation or synthesis, such as handling of missing summary statistics, or data conversions. | 7-9, Appendix |
|  | 13c | Describe any methods used to tabulate or visually display results of individual studies and syntheses. | 7-9, Appendix |
|  | 13d | Describe any methods used to synthesize results and provide a rationale for the choice(s). If meta-analysis was performed, describe the model(s), method(s) to identify the presence and extent of statistical heterogeneity, and software package(s) used. | 7-9, Appendix |
|  | 13e | Describe any methods used to explore possible causes of heterogeneity among study results (e.g. subgroup analysis, meta-regression). | 7-9, Appendix |
|  | 13f | Describe any sensitivity analyses conducted to assess robustness of the synthesized results. | 7-9, Appendix |
| Reporting bias assessment | 14 | Describe any methods used to assess risk of bias due to missing results in a synthesis (arising from reporting biases). | 7-9, Appendix |
| Certainty assessment | 15 | Describe any methods used to assess certainty (or confidence) in the body of evidence for an outcome. | 7-9, Appendix |
| **RESULTS** | | |  |
| Study selection | 16a | Describe the results of the search and selection process, from the number of records identified in the search to the number of studies included in the review, ideally using a flow diagram. | 10-11, Fig 1, eTab 2 |
|  | 16b | Cite studies that might appear to meet the inclusion criteria, but which were excluded, and explain why they were excluded. | 10-11, eTab 3 |
| Study characteristics | 17 | Cite each included study and present its characteristics. | 10-11, Tab 1 |
| Risk of bias in studies | 18 | Present assessments of risk of bias for each included study. | 10-11, eFig 4 |
| Results of individual studies | 19 | For all outcomes, present, for each study: (a) summary statistics for each group (where appropriate) and (b) an effect estimate and its precision (e.g. confidence/credible interval), ideally using structured tables or plots. | 11-13, Tab 1 |
| Results of syntheses | 20a | For each synthesis, briefly summarise the characteristics and risk of bias among contributing studies. | 11-13, Fig 2 |
|  | 20b | Present results of all statistical syntheses conducted. If meta-analysis was done, present for each the summary estimate and its precision (e.g. confidence/credible interval) and measures of statistical heterogeneity. If comparing groups, describe the direction of the effect. | 11-13, Fig 2, eFig 3 |
|  | 20c | Present results of all investigations of possible causes of heterogeneity among study results. | 14-15 |
|  | 20d | Present results of all sensitivity analyses conducted to assess the robustness of the synthesized results. | 15-18 |
| Reporting biases | 21 | Present assessments of risk of bias due to missing results (arising from reporting biases) for each synthesis assessed. | 15-18, eFig 4 |
| Certainty of evidence | 22 | Present assessments of certainty (or confidence) in the body of evidence for each outcome assessed. | 15-18 |
| **DISCUSSION** | | |  |
| Discussion | 23a | Provide a general interpretation of the results in the context of other evidence. | 19-22 |
|  | 23b | Discuss any limitations of the evidence included in the review. | 22-24 |
|  | 23c | Discuss any limitations of the review processes used. | 22-24 |
|  | 23d | Discuss implications of the results for practice, policy, and future research. | 24 |
| **OTHER INFORMATION** | | |  |
| Registration and protocol | 24a | Provide registration information for the review, including register name and registration number, or state that the review was not registered. | 4 |
|  | 24b | Indicate where the review protocol can be accessed, or state that a protocol was not prepared. | 4 |
|  | 24c | Describe and explain any amendments to information provided at registration or in the protocol. | 4 |
| Support | 25 | Describe sources of financial or non-financial support for the review, and the role of the funders or sponsors in the review. | 26 |
| Competing interests | 26 | Declare any competing interests of review authors. | 26 |
| Availability of data, code and other materials | 27 | Report which of the following are publicly available and where they can be found: template data collection forms; data extracted from included studies; data used for all analyses; analytic code; any other materials used in the review. | 26 |

The current checklist followed the latest PRISMA 2020 guideline [1].

**Reference**

1. Page, M.J.; McKenzie, J.E.; Bossuyt, P.M.; Boutron, I.; Hoffmann, T.C.; Mulrow, C.D.; Shamseer, L.; Tetzlaff, J.M.; Akl, E.A.; Brennan, S.E.; et al. The PRISMA 2020 statement: an updated guideline for reporting systematic reviews. *Bmj* **2021**, *372*, n71, doi:10.1136/bmj.n71.

**Table S2: the keyword and search result in the different databases**

| Database | Keyword* | Filter | Date | Result |
| --- | --- | --- | --- | --- |
| PubMed | (deep transcranial magnetic stimulation OR dTMS OR repetitive transcranial magnetic stimulation OR rTMS OR TMS OR non-invasive brain stimulation OR theta burst stimulation OR transcranial direct current stimulation OR TBS OR tDCS OR vagus nerve stimulation OR vagal nerve stimulation OR tVNS OR nVNS OR VNS OR static magnetic field stimulation) AND (skill OR professionalism OR skill acquisition) AND (surgery OR surgical OR surgeon) AND (random OR randomized OR randomised) | NA | 2021/4/19 | 21 |
| Embase | (deep transcranial magnetic stimulation OR dTMS OR repetitive transcranial magnetic stimulation OR rTMS OR TMS OR non-invasive brain stimulation OR theta burst stimulation OR transcranial direct current stimulation OR TBS OR tDCS OR vagus nerve stimulation OR vagal nerve stimulation OR tVNS OR nVNS OR VNS OR static magnetic field stimulation) AND (skill OR professionalism OR skill acquisition) AND (surgery OR surgical OR surgeon) AND (random OR randomized OR randomised) | NA | 2021/4/19 | 15 |
| ClinicalKey | (non-invasive brain stimulation) AND (surgery OR surgical OR surgeon) AND (random OR randomized OR randomised) | NA | 2021/4/19 | 9 |
| Cochrane CENTRAL | (deep transcranial magnetic stimulation OR dTMS OR repetitive transcranial magnetic stimulation OR rTMS OR TMS OR non-invasive brain stimulation OR theta burst stimulation OR transcranial direct current stimulation OR TBS OR tDCS OR vagus nerve stimulation OR vagal nerve stimulation OR tVNS OR nVNS OR VNS OR static magnetic field stimulation) AND (skill OR professionalism OR skill acquisition) AND (surgery OR surgical OR surgeon) AND (random OR randomized OR randomised) | NA | 2021/4/19 | 21 |
| ProQuest | (non-invasive brain stimulation) AND (surgery OR surgical OR surgeon) AND (skill) AND (random OR randomized OR randomised) | NA | 2021/4/19 | 4930 |
| ScienceDirect | (non-invasive brain stimulation) AND (surgery OR surgical OR surgeon) AND (skill) AND (random OR randomized OR randomised) | Research article | 2021/4/19 | 757 |
| Web of Science | (deep transcranial magnetic stimulation OR dTMS OR repetitive transcranial magnetic stimulation OR rTMS OR TMS OR non-invasive brain stimulation OR theta burst stimulation OR transcranial direct current stimulation OR TBS OR tDCS OR vagus nerve stimulation OR vagal nerve stimulation OR tVNS OR nVNS OR VNS OR static magnetic field stimulation) AND (skill OR professionalism OR skill acquisition) AND (surgery OR surgical OR surgeon) AND (random OR randomized OR randomised) | NA | 2021/4/19 | 5 |
| ClinicalTrials.gov | (non-invasive brain stimulation) AND (surgery OR surgical OR surgeon) AND (skill) AND (random OR randomized OR randomised) | NA | 2021/4/19 | 0 |

*: in order to try to find as many potential eligible articles as possible, we also applied other keywords of non-invasive brain stimulation method in our keyword.

Abbreviation: NA: not applied

**Table S3: Excluded studies and reason**

| Reason | Numbers | References |
| --- | --- | --- |
| Duplicate sample source with the included studies | 3 | [1-4] |
| Not related to surgical skill training | 2 | [5,6] |

**References:**

1. Ciechanski, P.; Cheng, A.; Damji, O.; Lopushinsky, S.; Hecker, K.; Jadavji, Z.; Kirton, A. Effects of transcranial direct-current stimulation on laparoscopic surgical skill acquisition: a randomized clinical trial. *Brain stimulation* **2017**, *10*, 534.

2. Ciechanski, P.; Hecker, K.; Wilson, B.; Williams, C.; Lopushinsky, S.; Anderson, S.; Cheng, A.; Kirton, A. Neural correlates of transcranial direct-current stimulation enhanced surgical skill learning. *Brain stimulation* **2019**, *12*, 398.

3. Appelbaum, L.G.; Deng, Z.D.; Palmer, H.; Beynel, L.; Watts, A.; Young, J.R.; Lisanby, S.H.; Migaly, J.; Cox, M.L. Proceedings 2 Transcranial Direct Current Stimulation to Enhance Laparoscopic Technical Skill Learning: A Preregistered Randomized Controlled Trial. *Brain stimulation* **2019**, *12*, e57-e59.

4. Patel, R.; Singh, H.; Ashcroft, J.; Woods, A.J.; Darzi, A.; Leff, D.R. Dataset of prefrontal transcranial direct-current stimulation to improve early surgical knot-tying skills. *Data Brief* **2021**, *35*, 106905, doi:10.1016/j.dib.2021.106905.

5. Focke, J.; Kemmet, S.; Krause, V.; Keitel, A.; Pollok, B. Cathodal transcranial direct current stimulation (tDCS) applied to the left premotor cortex (PMC) stabilizes a newly learned motor sequence. *Behavioural brain research* **2017**, *316*, 87-93, doi:10.1016/j.bbr.2016.08.032.

6. Patel, R.; Wilkinson, A.; Singh, H.; Leff, D. P99 Attenuation of perceived temporal demand during surgical tasks with transcranial direct-current stimulation. *Clinical Neurophysiology* **2020**, *131*, e68.
